# Comparison of quality of life between patients undergoing trans-oral endoscopic thyroid surgery and conventional open surgery

**DOI:** 10.1186/s12893-020-0685-3

**Published:** 2020-01-29

**Authors:** Pornthep Kasemsiri, Srongpaun Trakulkajornsak, Piyapong Bamroong, Kanokkarn Mahawerawat, Patorn Piromchai, Teeraporn Ratanaanekchai

**Affiliations:** 1 Skull Base Surgery Unit, Department of Otorhinolaryngology, Srinagarind Hospital, Faculty of Medicine at the Khon Kaen University, Khon Kaen, 40002 Thailand; 2 Department of Otorhinolaryngology, Mukdahan Hospital, Mukdahan, Thailand; 3 Khon Kaen Head and Neck Oncology Research, Khon Kaen, Thailand; 4 Srinagarind Minimally Invasive Surgery Center of Excellence, Khon Kaen, Thailand

**Keywords:** Thyroidectomy, Endoscopy, Quality of life, Scarring

## Abstract

**Background:**

Trans-oral endoscopic thyroidectomy allows obviating scar of the neck that expects to gain quality of life (QOL). However, the benefit of the QOL from this technique has not been adequately investigated, therefore, this study compared the QOL outcomes, including cosmetic outcomes, between thyroidectomy by trans-oral endoscopy and conventional open surgery.

**Methods:**

A study was conducted from January 30, 2017 to November 10, 2018. Thirty-two and 38 patients underwent trans-oral endoscopic thyroid surgery and conventional open surgery, respectively. Their quality of life was evaluated at 2, 6, and 12 weeks postoperatively using a thyroid surgery-specific questionnaire and a 36-item short-form questionnaire.

**Results:**

Trans-oral endoscopic group, patients were younger and presented with smaller thyroid nodules (*p* < 0.05). Regarding surgical outcomes, there were no statistically significant differences between the two groups. Mean operative time was significantly longer in the trans-oral endoscopic group (*p* < 0.05). The quality of life parameters in the trans-oral endoscopic group was significantly better than in the conventional surgery group (*p* < 0.05). These parameters included reduction of physical activity, psychosocial impairment, the role of physic, and emotion at 2 weeks after surgery; swallowing impairment, psychosocial impairment, the role of physic, social function and mental health 6 weeks after surgery; tingling and feeling of vitality at 12 weeks after surgery. Cosmetic outcomes and overall satisfaction were significantly better in the trans-oral endoscopic group than in the conventional surgery group at all of our follow up times (*p* < 0.05).

**Conclusions:**

The trans-oral endoscopic approach allows real scarless on the skin with better cosmetic and QOL outcomes.

**Trial registration:**

This trial was retrospectively registered at the ClinicalTrial.gov (NCT03048539), registered on 4 March 2017.

## Introduction

The quality of life (QOL) of patients with post thyroidectomy scars is impaired [1] after conventional or traditional thyroidectomy with typically approached through a transverse skin incision in the anterior neck [2]. This technique causes scar formation in an obvious area of the neck. Thus, various techniques have been developed to avoid the scarring on the neck. Endoscopic thyroidectomy, first introduced by Huscher et al. [3], makes possible a variety of routes to the thyroid. These include a trans-axillary approach [4, 5], trans-anterior chest wall approach [6], trans-breast approach [7–9], and retro-auricular approach [10]. However, all still cause some scarring. Therefore, a trans-oral endoscopic thyroidectomy approach has been developed, which completely obviates scarring of the skin. This approach uses laparoscopic instruments via the oral vestibule, through the premandibular space with CO_2_ insufflation. There is increasing evidence that trans-oral endoscopic thyroidectomy causes less tissue damage, is safer and cosmetically satisfactory. However, the QOL outcomes among patients undergoing this form of surgery have not been adequately investigated; thus, we developed this study to analyze and compare functional parameters, including QOL and cosmetic outcomes.

## Materials and methods

### Study design

The prospective cross-sectional observational study was developed to compare QOL after surgery between patients undergoing endoscopic thyroidectomy and those who received conventional open thyroidectomy. All patients were informed of the surgical options and specified which was their preferred option. This study was conducted in two centers between January 30, 2017 and November 10, 2018. Patients between the ages of 18 and 70 years, who presented with a single thyroid nodule less than 5 cm in diameter, were regarded as eligible for inclusion. Exclusion criterias included patients with any of the following; previous neck surgery; suspected malignancy following investigations including fine-needle aspiration, ultrasound, CT or MRI; suspected metastatic lymphadenopathy; converted technique from endoscopic approach to open approach during surgery.

Regarding sample size, the formula of longitudinal data analysis [11] was applied that allowed the sample size of 38 patients per group with 10% of the patients lost to follow up. This sample size was deemed appropriate considering 95% confidence intervals, 2% error, the correlation coefficient of the repeated measurements of 0.5, the standard deviation of general health score in the 36-Item Short-Form Health Survey (SF-36) of 10.8 and mean difference general health score in SF-36 of 6 that estimated from the study of Huang JK et al. [4].

### Surgical technique

The trans-oral endoscopic approach used was identical to that reported in a previous study [12]. the patient was placed in a supine position with neck extension under general anesthesia and nasotracheal intubation. Antibiotic prophylaxis was administered before the incision was made. Two 5 mm laparoscopic ports were placed at the junction between the canine and the first premolar tooth. Another 10 mm port was placed in the midline. These ports were inserted under the lower lip at the vestibular region. A 10-mm 30° lens allowed the surgical view. The surgical corridor was created down to the sternal notch with the lateral border at the sternocleidomastoid muscles. The strap muscles were separated in the midline and retracted laterally. The thyroid isthmus was divided in the midline and then the superior pole was dissected and the superior thyroid vessels controlled. Dissection of the thyroid lobe with preservation of the recurrent laryngeal nerve (RLN) was performed downwards and parallel to the trachea. Finally, the thyroid lobe was removed via a 10-mm midline port. Suturing of the strap muscles in the midline and suturing of the vestibular incision in the oral cavity was performed. No surgical wound was visible on the skin.

For conventional open thyroidectomy, the patient was placed in a supine position with neck extension under orotracheal intubation. A 5- to 10-cm transverse collar incision was made two fingerbreadths above the sternal notch. A subplatysmal flap was elevated up to the thyroid cartilage and down to the sternal notch. The strap muscles were separated in the midline to expose the thyroid gland. Superior thyroid vessels were identified and ligated. Dissection of the thyroid lobe with the preservation of the RLN was performed. The middle thyroid vein and inferior thyroid vessels were controlled. The thyroid lobe and isthmus were removed. The surgical wound was closed layer by layer. Suturing of the wound on the skin was done in two techniques, simple sutures using nylon (5–0) and subcuticular suturing using poliglecaprone 25 (4–0).

### QOL assessment

The QOL was assessed using a questionnaire specifically for outcomes after thyroid surgery and SF-36 questionnaire at 2, 6 and 12 weeks postoperatively. The specific questionnaire included 10 items. Overall satisfaction and VAS pain score at the operation site was graded 0 to 10. Neck movement, shoulder movement, voice, and swallowing were evaluated according to the subjective assessment of patients with grading as never experiencing any difficulty in doing these: 0, almost never (occasionally): 1, sometimes: 2, almost always: 3, always: 4. The tingling sensation and paresthesia change were assessed by the existence of stabbing pain and numbness in the neck and chin areas with grading as no pain or other abnormal sensation: 0, minimal: 1, moderate: 2, severe: 3. Physical activity and psychosocial impairments were assessed by comparing the pre- and postoperative status with grading as no impairment: 0, almost never (occasionally): 1, sometimes: 2, almost always: 3, always: 4. The patient’s general QOL was assessed using the SF-36 questionnaire. This covers eight parameters including physical functioning, role-physical, bodily pain, general health, vitality, social functioning, role-emotional, and mental health. The higher the score, the better the outcome in each case. Multiple comparisons of numeric parameters were analyzed by Generalized Estimating Equation with Bonferroni-adjusted *p*-value whereas ordinal parameters were analyzed with ordinal logistic regression adjusted cluster effect. A value of *p* < 0.05 was considered statistically significant. The study was approved by the Local Ethics Committee (HE591505). In addition, this study was registered with the ClinicalTrial.gov (NCT03048539).

## Results

Seventy-six patients underwent thyroid lobectomy: 38 selected the conventional open approach whereas 32 patients opted for trans-oral endoscopic thyroidectomy. The other six patients chose another endoscopic approach. Therefore, data from 70 patients were analyzed to compare QOL between those undergoing trans-oral endoscopic thyroid lobectomy and those submitting to conventional open surgery.

In both groups, most patients were female presenting with similar clinicopathology. Patients in the trans-oral endoscopic group were significantly younger and presented with significantly smaller thyroid nodules (Table 1). In terms of surgical outcomes, there were no statistically significant differences between the two groups in blood loss, hospital stay time or complications. However, mean operative time was significantly longer in the trans-oral endoscopic group (Table 1).

**Table 1:** Demographic data

Characteristics	Trans-oral endoscopic thyroidectomy	Open thyroidectomy	*P* -Value
Gender: n (%)			
Male	0	4 (10.5) (95% CI: 4.2–24.1)	*P* > 0.05
Female	32 (100) (95% CI: 89.3–100)	34 (89.5) (95% CI: 7.6–95.8)	
Age (Mean ± SD; year)	38.3 ± 11.3 (95% CI: 34.2–42.4)	46.7 ± 10.9 (95% CI: 43.1–50.3)	*P* < 0.05
Pathology: n (%)			
Benign nodule	31 (96.9) (95% CI: 84.3–99.5)	38 (100) (95% CI: 90.8–100)	*P* > 0.05
Micropapillary carcinoma	1 (3.1) (95% CI: 0.6–15.7)	0	
Nodule size (Mean ± SD; cm)	2.8 ± 1.2 (95% CI: 2.4–3.2)	3.4 ± 1.2 (95% CI: 3.0–3.8)	*P* < 0.05
Operative time (Mean ± SD; min)	123.8 ± 26.3 (95% CI: 114.3–133.3)	84.1 ± 35.5 (95% CI: 72.4–95.8)	*P* < 0.05
Blood loss (Mean ± SD; cc)	23.9 ± 46.2 (95% CI: 7.2–40.6)	24.2 ± 29.0 (95% CI: 14.7–33.7)	*P* > 0.05
Hospital stay (Mean ± SD; day)	3.3 ± 0.7 (95% CI: 3.1–3.6)	3.4 ± 0.8 (95% CI: 3.1–3.7)	*P* > 0.05
Complication: n (%)			
Vocal cord paresis	3 (9.3) (95% CI: 3.2–24.2)	1 (2.6) (95% CI: 0.4–13.4)	*P* > 0.05
Vocal cord paralysis	1 (3.1) (95% CI: 0.6–15.7)	1 (2.6) (95%CI: 0.4–13.4)	*P* > 0.05
Surgical site infection	0	0	*P* > 0.05

QOL was evaluated using two questionnaires. The thyroid surgery-specific questionnaire showed that some parameters in the trans-oral endoscopic group were better than in the conventional open surgery group (Table 2). These parameters included significant decreases in physical reduction and psychosocial impairment at 2 weeks after surgery, significant decreases in swallowing and psychosocial impairment at 6 weeks after surgery, and a significant decrease in tingling at 12 weeks after surgery. Furthermore, cosmetic and overall satisfaction were significantly greater in the trans-oral endoscopic group at all of our follow up periods (Table 2).

**Table 2:** Comparison of quality-of-life parameters between the group undergoing trans-oral endoscopic thyroidectomy and the group undergoing conventional open thyroidectomy. This table is based on the specific thyroid surgery-related QOL questionnaire

Specific thyroid surgery-related QOL parameter and grade	Postoperative 2 weeks			Postoperative 6 weeks			Postoperative 12 weeks		
	Trans-oral endoscopic thyroidectomy	Open thyroidectomy	*P* value	Trans-oral endoscopic thyroidectomy	Open thyroidectomy	*P* value	Trans-oral endoscopic thyroidectomy	Open thyroidectomy	*P* value
Numbness N (%): 0	10 (31.3)	25 (65.8)	0.053	15 (46.9)	26 (68.4)	0.398	18 (56.3)	25 (69.4)	0.819
1	12 (37.5)	6 (15.8)		11 (34.4)	5 (13.2)		9 (28.1)	7 (19.4)	
2	5 (15.6)	2 (5.3)		1 (3.1)	4 (10.5)		2 (6.3)	2 (5.6)	
3	5 (15.6)	5 (13.2)		5 (15.6)	3 (7.9)		3 (9.4)	2 (5.6)	
Tingling N (%): 0	20 (62.5)	12 (31.6)	0.060	19 (59.4)	13 (34.2)	0.052	22 (68.8)	13 (36.1)	0.009*
1	7 (21.9)	15 (39.5)		10 (31.3)	13 (34.2)		9 (28.1)	15 (41.7)	
2	3 (9.4)	8 (21.1)		1 (3.1)	9 (23.7)		0 (0.0)	4 (11.1)	
3	2 (6.3)	3 (7.9)		2 (6.3)	3 (7.9)		1 (3.1)	4 (11.1)	
Cosmetic N (%): 4	28 (87.5)	10 (26.3)	< 0.001*	24 (75.0)	10 (26.3)	< 0.001*	30 (93.8)	12 (33.3)	< 0.001*
3	3 (9.4)	7 (18.4)		6 (18.8)	10 (26.3)		1 (3.1)	12 (33.3)	
2	1 (3.1)	14 (36.8)		1 (3.1)	15 (39.5)		0 (0.0)	8 (22.2)	
1	0 (0.0)	7 (18.4)		0 (0.0)	3 (7.9)		0 (0.0)	2 (5.6)	
0	0 (0.0)	0 (0.0)		1 (3.1)	0 (0.0)		1 (3.1)	2 (5.6)	
Voice impairment N (%): 0	24 (75.0)	20 (52.6)	0.404	26 (81.3)	19 (50.0)	0.063	29 (90.6)	26 (72.2)	0.269
1	0 (0.0)	3 (7.9)		1 (3.1)	3 (7.9)		1 (3.1)	5 (13.9)	
2	1 (3.1)	2 (5.3)		2 (6.3)	10 (26.3)		1 (3.1)	4 (11.1)	
3	1 (3.1)	5 (13.2)		1 (3.1)	4 (10.5)		0 (0.0)	0 (0.0)	
4	6 (18.8)	8 (21.1)		2 (6.3)	2 (5.3)		1 (3.1)	1 (2.8)	
Swallowing impairment N (%): 0	19 (59.4)	18 (47.4)	0.281	24 (75.0)	19 (50.0)	0.036*	27 (84.4)	26 (72.2)	0.508
1	4 (12.5)	0 (0.0)		2 (6.3)	3 (7.9)		2 (6.3)	2 (5.6)	
2	4 (12.5)	9 (23.7)		5 (15.6)	6 (15.8)		3 (9.4)	4 (11.1)	
3	3 (9.4)	1 (2.6)		0 (0.0)	1 (2.6)		0 (0.0)	1 (2.8)	
4	2 (6.3)	10 (26.3)		1 (3.1)	9 (23.7)		0 (0.0)	3 (8.3)	
Neck movement impairment N (%): 0	6 (18.8)	10 (26.3)	> 0.999	12 (37.5)	15 (39.5)	> 0.999	21 (65.6)	18 (50.0)	0.622
1	6 (18.8)	3 (7.9)		10 (31.3)	5 (13.2)		5 (15.6)	8 (22.2)	
2	8 (25.0)	9 (23.7)		5 (15.6)	8 (21.1)		3 (9.4)	6 (16.7)	
3	3 (9.4)	4 (10.5)		1 (3.1)	3 (7.9)		2 (6.3)	0 (0.0)	
4	9 (28.1)	12 (31.6)		4 (12.5)	7 (18.4)		1 (3.1)	4 (11.1)	
Shoulder movement impairment N (%): 0	30 (93.8)	36 (94.7)	> 0.999	30 (93.8)	33 (86.8)	> 0.999	32 (100.0)	34 (94.4)	NA
1	2 (6.3)	0 (0.0)		1 (3.1)	3 (7.9)		0 (0.0)	1 (2.8)	
2	0 (0.0)	1 (2.6)		1 (3.1)	2 (5.3)		0 (0.0)	0 (0.0)	
3	0 (0.0)	0 (0.0)		0 (0.0)	0 (0.0)		0 (0.0)	0 (0.0)	
4	0 (0.0)	1 (2.6)		0 (0.0)	0 (0.0)		0 (0.0)	1 (2.8)	
Physical activity reduction N (%): 0	22 (68.8)	11 (29.0)	0.001*	27 (84.4)	26 (68.4)	0.303	29 (90.6)	29 (80.6)	0.620
1	3 (9.4)	1 (2.6)		3 (9.4)	4 (10.5)		3 (9.4)	3 (8.3)	
2	2 (6.3)	10 (26.3)		1 (3.1)	4 (10.5)		0 (0.0)	2 (5.6)	
3	3 (9.4)	3 (7.9)		1 (3.1)	3 (7.9)		0 (0.0)	1 (2.8)	
4	2 (6.3)	13 (34.2)		0 (0.0)	1 (2.6)		0 (0.0)	1 (2.8)	
Psychosocial impairment N (%): 0	28 (87.5)	17 (44.7)	< 0.001*	29 (90.6)	25 (65.8)	0.028*	32 (100.0)	28 (77.8)	NA
1	3 (9.4)	4 (10.5)		3 (9.4)	4 (10.5)		0 (0.0)	2 (5.6)	
2	0 (0.0)	3 (7.9)		0 (0.0)	1 (2.6)		0 (0.0)	2 (5.6)	
3	1 (3.1	1 (2.6)		0 (0.0)	2 (5.3)		0 (0.0)	3 (8.3)	
4	0 (0.00)	13 (34.2)		0 (0.0)	6 (15.8)		0 (0.0)	1 (2.8)	
VAS Pain score (0–10) Mean (SD)	1.6 (2.0)	1.6 (2.3)	> 0.999	0.6 (1.5)	1.3 (1.9)	0.298	0.3 (1.0)	0.6 (1.5)	> 0.999
Overall satisfaction (10–0) Mean (SD)	8.9 (1.3)	7.3 (2.2)	< 0.001*	9.4 (0.7)	8.1 (1.8)	0.001*	9.8 (0.4)	8.9 (1.7)	0.022*

* *p* < 0.05: statistically significance

All parameters from the SF-36 questionnaire were better in the trans-oral endoscopic group than in the conventional open-surgery group (Table 3). Six parameters stood out as statistically significantly different. These were the role of physic and emotion at 2 weeks after surgery, the role of physic, social function and mental health at 6 weeks after surgery, and vitality at 12 weeks after surgery.

**Table 3:** Comparison of quality-of-life parameters between the group undergoing trans-oral endoscopic thyroidectomy and the group undergoing conventional open thyroidectomy. This table is based on the SF-36 questionnaire

SF 36	Post op 2 weeks			Post op 6 weeks			Post op 12 weeks		
	Endo (n:32)	Open (n:38)	*P*-value	Endo (n:32)	Open (n:38)	*P*-value	Endo (n:32)	Open (n:38)	*P*-value
Physical function	83.9 (25.9)	74.4 (24.7)	0.244	93.8 (20.1)	81.3 (22.1)	0.064	95.3 (19.5)	87.2 (24.4)	0.378
Role physic	85.2 (28.3)	49.3 (44.5)	< 0.001*	87.5 (28.4)	59.2 (37.4)	0.001*	100.0 (0.0)	84.5 (31.4)	0.108
Bodily pain	78.8 (29.4)	77.5 (30.0)	> 0.999	91.1 (23.9)	81.3 (22.1)	0.351	92.4 (24.3)	80.8 (28.6)	0.188
General health	74.1 (25.9)	72.0 (25.6)	> 0.999	83.4 (21.4)	73.5 (21.7)	0.235	81.0 (23.6)	73.3 (25.3)	0.458
Vitality	65.9 (34.0)	58.9 (28.1)	0.928	78.1 (32.4)	61.6 (27.6)	0.053	82.8 (25.6)	64.0 (29.1)	0.027*
Social function	84.9 (25.5)	71.4 (36.3)	0.140	95.3 (19.5)	76.3 (33.2)	0.016*	90.6 (26.8)	86.1 (25.8)	> 0.999
Role emotion	88.5 (26.2)	65.8 (43.5)	0.004*	95.3 (19.5)	82.5 (29.8)	0.212	92.2 (25.7)	88.3 (27.5)	> 0.999
Mental health	72.2 (28.7)	69.5 (25.8)	> 0.999	88.3 (21.8)	69.6 (23.9)	0.004*	87.2 (24.2)	82.2 (23.5)	> 0.999

* *p* < 0.05: statistically significance

## Discussion

The use of endoscopic surgery has been growing in management of thyroid neoplasm. Endoscopic thyroidectomy is a safe, minimally invasive technique producing good cosmetic satisfaction and improved postoperative QOL outcomes. The trans-oral endoscopic route leaves no scar on the skin after thyroid surgery. Younger patients are particularly concerned about scarring. In our series, younger patients were significantly more likely to choose the trans-oral endoscopic technique than the conventional open surgery technique (*p* < 0.05) for this reason. Regarding feasibility and safety, the trans-oral endoscopic approach did not significantly differ from the conventional open surgery approach. Four patients in the trans-oral endoscopic group experienced postoperative vocal cord malfunction as against two patients in the conventional open surgery group. All three patients with vocal cord paresis recovered within 6 weeks. Vocal cord paresis in the trans-oral endoscopic group may be caused by heat from the ultrasonic scalpel. Regular pauses while using the ultrasonic scalpel near the recurrent laryngeal nerve (RLN) are therefore recommended. Furthermore, intraoperative neural monitoring (IONM) was introduced to prevent RLN injuries in transoral thyroidectomy. Wang et al. [13] reported no patients experienced transient or permanent RLN palsy after implement of IONM. However, the systematic review showed no statistically significant differences between visualization alone and visualization plus IONM [14]; thus, it is not considered as a “standard of care” [15]. Although IONM seems like defensive medicine, it allows the benefits including identifying nerve and preventing misidentification of any cord-like structure [16], and allowing information about the functional status of visually intact nerves [17]. Regarding volume of blood loss, it was similar between the groups. However, the endoscopic procedure took significantly longer, as noted in previous studies [4, 18]. The longer operative time may increase the risk of complications and morbidity due to longer exposure time to general anesthesia. Greater surgical skill and experience may help to reduce the time needed [19, 20].

Numbness after surgery is a common complaint affecting the QOL of patients. It results from injury to the cutaneous nerve. The trans-oral endoscopic approach requires a corridor from the chin to the neck. Three ports were used via the lower vestibule that may cause injury to a branch of the mental nerve. Therefore, in the trans-oral endoscopic group, numbness was felt on the chin and lower lip, whereas the conventional open surgery group reported numbness on the neck. However, the incidence of numbness did not significantly differ between the groups (*p* > 0.05). There was some concern that fibrosis of the neck due to the creation of the corridor may disturb swallowing and neck movement. However, these parameters were similar in both groups (*p* > 0.05), except that the endoscopic group experienced significantly less swallowing impairment at 6 weeks after surgery (*p* = 0.036). Another complaint concerned a pulling sensation that may result from subplatysmal dissection and subsequent fibrosis after creating the corridor. Bakkar et al. [21] reported that some patients with trans-oral endoscopic thyroidectomy had a long-lasting (up to 6 months) bothersome pulling sensation along the surgical tract. We found this problem occurred within only the first 2 weeks and resolved within 6 weeks after surgery because our patients were educated about the importance of neck exercise as soon as possible after surgery. Such exercise might ease postoperative fibrosis adhesion of the neck [22].

Rapid recovery after surgery allowing an early return to work is a common wish of patients. At 2 weeks after surgery, the post-operative physical reduction in specific thyroid surgery questionnaire was significantly lower in the transoral endoscopic group (*p* = 0.001). This finding was related to other parameters in SF-36, including role of physic (at 2 and 6 weeks after surgery), and vitality (at 12 weeks after surgery), that were provided significantly higher score in the transoral endoscopic group (*p* < 0.05). These findings are meaningfully allowed that patients in the transoral endoscopic group might earlier return to their work than conventional open group. It would gain the benefit of QOL in an early period. Regarding specific thyroid surgery questionnaire, cosmetic parameter was significantly higher in transoral endoscopic group (*p* < 0.001). This finding may be associated with significantly lower psychosocial impairment parameter in transoral endoscopic group, also (*p* < 0.001 and *p* = 0.028 at 2 weeks and 6 weeks after surgery, respectively). At 12 weeks after surgery, patients had not psychosocial impairment in transoral endoscopic group. Furthermore, social function, role emotion, and mental health were significantly better in the transoral endoscopic group (*p* < 0.05 at 2 weeks and 6 weeks after surgery, respectively). These findings supported that the cosmetic outcomes and QOL in transoral endoscopic group better than conventional open group. Although postoperative pain seemed similar in both groups, the overall satisfaction in transoral endoscopic group was superior to conventional open group.

This observational study is the first to compare the QOL between conventional open surgery and trans-oral endoscopic thyroidectomy in generic and specific health (SF-36 and thyroid surgery-specific questionnaire, respectively). The latter leaves no visible scar, leading to superior QOL outcomes. However, this study has some limitations including small sample size, short-term follow-up, and surgeon’s learning curve. Furthermore, almost our patients presented with a benign nodule; therefore, further study should be additionally designed to compare QOL between conventional open surgery and trans-oral endoscopic thyroidectomy in the malignant nodule. Because malignant patients will be treated with more aggressive surgical approaches including total thyroidectomy with or without central neck dissection; thus, the QOL results may be difference from patients with benign nodule. Recently, trans-oral endoscopic technique has been developed to act as the alternative option of conventional open technique that allows feasibility and safety for total thyroidectomy with central neck dissection [14]. Additionally, the issue of prophylactic central neck dissection in malignant thyroid cancer is still controversial due to allowing more surgical complications [23] that may lead to poor QOL; however, the comparing of QOL between total thyroidectomy with and without prophylactic central neck dissection has never been reported in previous study. Therefore, further study should be investigated in this issue.

## Conclusion

Safety, feasibility, and patient satisfaction of trans-oral endoscopic thyroidectomy were established. The patients who underwent this technique had a superior perception of their mental health, physical function and participation in social activity. Trans-oral endoscopic thyroidectomy achieves excellent QOL outcomes after surgery.

## Data Availability

The datasets used and/or analyzed during the current study are available from the corresponding author on reasonable request.

## Abbreviations

CT: Computed Tomography
IONM: Intraoperative neural monitoring
MRI: Magnetic resonance imaging
QOL: Quality of life
RLN: Recurrent laryngeal nerve
SF-36: 36-Item Short-Form Health Survey
